# The SmpB-tmRNA Tagging System Plays Important Roles in *Streptomyces coelicolor* Growth and Development

**DOI:** 10.1371/journal.pone.0004459

**Published:** 2009-02-12

**Authors:** Chunzhong Yang, John R. Glover

**Affiliations:** Department of Biochemistry, University of Toronto, Toronto, Ontario, Canada; Baylor College of Medicine, United States of America

## Abstract

The *ssrA* gene encodes tmRNA that, together with a specialized tmRNA-binding protein, SmpB, forms part of a ribonucleoprotein complex, provides a template for the resumption of translation elongation, subsequent termination and recycling of stalled ribosomes. In addition, the mRNA-like domain of tmRNA encodes a peptide that tags polypeptides derived from stalled ribosomes for degradation. *Streptomyces* are unique bacteria that undergo a developmental cycle culminating at sporulation that is at least partly controlled at the level of translation elongation by the abundance of a rare tRNA that decodes UUA codons found in a relatively small number of open reading frames prompting us to examine the role of tmRNA in *S. coelicolor*. Using a temperature sensitive replicon, we found that the *ssrA* gene could be disrupted only in cells with an extra-copy wild type gene but not in wild type cells or cells with an extra-copy mutant tmRNA (tmRNA^DD^) encoding a degradation-resistant tag. A cosmid-based gene replacement method that does not include a high temperature step enabled us to disrupt both the *ssrA* and *smpB* genes separately and at the same time suggesting that the tmRNA tagging system may be required for cell survival under high temperature. Indeed, mutant cells show growth and sporulation defects at high temperature and under optimal culture conditions. Interestingly, even though these defects can be completely restored by wild type genes, the *ΔssrA* strain was only partially corrected by tmRNA^DD^. In addition, wildtype tmRNA can restore the hygromycin-resistance to Δ*ssrA* cells while tmRNA^DD^ failed to do so suggesting that degradation of aberrant peptides is important for antibiotic resistance. Overall, these results suggest that the tmRNA tagging system plays important roles during *Streptomyces* growth and sporulation under both normal and stress conditions.

## Introduction

In bacteria, stalled ribosomes can be released from mRNAs by the action of a specialized bifunctional RNA, called tmRNA, that acts as both an alanyl-tRNA and an mRNA encoding a short polypeptide tag [1]–[3]. Stalled ribosomes can be released via peptidyl transfer to the alanylated-tRNA-like domain of tmRNA followed by resumption of elongation on the mRNA-like domain. After translation termination, polypeptides bearing the C-terminal extension encoded by tmRNA are targeted for degradation, primarily by the ATP-dependent ClpXP protease [4]. In addition, tmRNA stimulates the cleavage of defective messages [5]. So far “broken” mRNAs lacking termination codons [6], [7], and mRNAs possessing inefficient termination codons [8], certain C-terminal codons [9], or containing rare codons [10] have been shown to elicit tmRNA tagging.

In *E. coli* [11] and *Bacillus subtilis* [12] tmRNA is not essential, but deletion of the *ssrA* gene that encodes tmRNA results in slow, temperature-sensitive growth. In the cyanobacterium, *Synechocystis* [13], and *E. coli* [14], elimination of tmRNA causes increased sensitivity to translation inhibitors. In *Neisseria gonorrhoeae ssrA* is essential [15]. Interestingly, in this organism viability is restored by expression of tmRNA encoding a tag with two C-terminal Ala residues changed to Asp. Since these substitutions alter residues critical for the recognition of tmRNA-tagged polypeptides by the Clp proteases it is thought that ribosome recycling is the essential function of tmRNA in *N. gonorrhoeae* whereas efficient degradation of tagged polypeptides is dispensable.

We undertook a study of *ssrA* function in *Streptomyces coelicolor* for several reasons. First, members of the genus *Streptomyces* have unusually G+C-rich genomes (72% in the case of *S. coelicolor*) raising the possibility that some mRNAs may have significant secondary structure that might inhibit translation elongation. Second, *Streptomyces* spp. undergo a developmental program when cultured on solid medium consisting of a substrate mycelial phase followed by the formation of aerial mycelia that septate to form spores. One of the genes required for the transition from substrate to aerial hyphae, *bldA*, encodes a tRNA that decodes the rare UUA codon and whose abundance is enhanced as cells enter stationary phase [16], [17]. UUA codons are present in restricted number of *S. coelicolor* ORFs [18] including *actII-ORF4* controlling actinorhidin production (blue pigment) [19], *redZ* controlling undecylprodigiosin production (red pigment) [20], and *adpA* (*bldH*) controlling morphogenesis of aerial hyphae [21]. Since stalling of ribosomes at rare codons results in *ssrA*-mediated tagging in other organisms [10], it is reasonable to speculate that part of the mechanism of postranscriptional control of the abundance of proteins translated from UUA-containing mRNAs might involve tmRNA-mediated tagging and subsequent degradation of truncated polypeptides during vegetative growth when the *bldA*-encoded tRNA is scarce. Third, *Streptomyces* spp. are used as hosts for the heterologous expression of foreign proteins [22]–[24] whose mRNAs could be subject to various defects in translation efficiency that would limit the accumulation of recombinant proteins. The tmRNA-tagging system might therefore be exploited to pinpoint sites on target mRNAs prone to ribosome stalling as means of optimizing the performance of expression systems.

To explore the function of *ssrA* in *S. coelicolor*, we used a commonly employed strategy in which cells were transformed with a temperature sensitive replicon and then exposed to high temperature to lose the plasmid. We were able to obtain disruptions in the *ssrA* gene only in strains carrying an extra copy of the wild type gene at a different locus. However using a cosmid-based disruption method we obtained insertional mutants of not only the *ssrA* gene, but also *smpB* gene and both genes simultaneously. We find that Δ*ssrA* and Δ*smpB* strains show apparent growth and sporulation defects at 30°C that were enhanced at 39°C. The slow growth phenotype and profound high-temperature sporulation defect in the *ΔssrA* strain explain why we were unable to recover mutants using the temperature sensitive plasmid. In addition, Δ*ssrA* and Δ*smpB* mutants are much more sensitive to hygromycin than wild type strain. Expression of a modified tmRNA encoding a degradation-resistant tag only partially restored high-temperature growth and sporulation in Δ*ssrA* strains but not hygromycin resistance suggesting that promoting the degradation of tagged proteins is an important role for tmRNA in cells challenged with a translation inhibitor. While this work was in progress a similar set of experiments investigating the role of tmRNA in the closely allied species *S. lividans* was published [25]. Our work corroborates many of the observations made but differs in some results permitting us to deepen the interpretation of the observations.

## Results

### Analysis of protein tagging by mutant tmRNA

**Figure 1:**
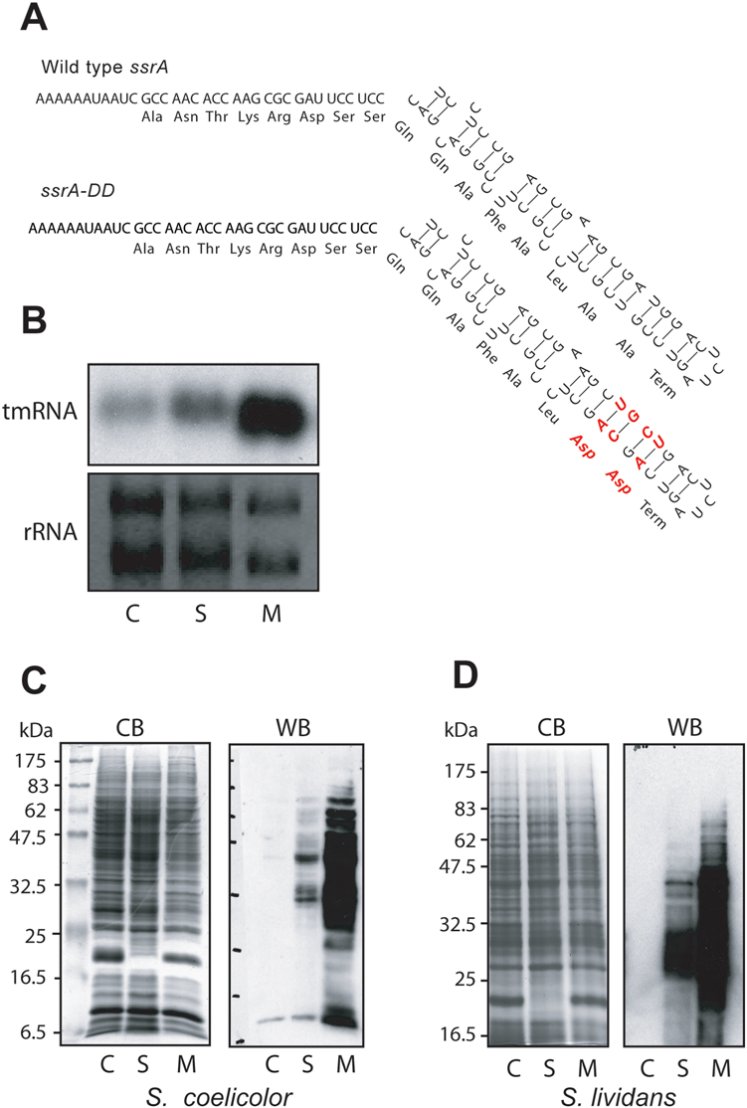
Construction of *ssrA-DD* mutant of *S. coelicolor ssrA* gene and detection of tagged proteins. A. Detail of the sequence and proposed secondary structure of the tag-coding sequence of *S. coelicolor* tmRNA. Bases in red in *ssrA-DD* were altered by mutagenesis. B. Northern blot analysis of total RNA isolated from empty vector transformed cells (C = control), and cells expressing tmRNA from a single-copy (S) and multi-copy (M) plasmid carrying *ssrA-DD*. Ethidium bromide stained ribosomal RNA (rRNA) is shown as a loading control. Note that the probe is generated from full-length *ssrA* DNA and does not distinguish between the wild type and mutant tmRNA. C. Western blot detection of tmRNA^DD^ tagging in *S. coelicolor*. Total proteins from strains analyzed in panel B were separated by SDS-PAGE and either stained with Coomassie blue (CB) or transferred to PVDF membranes and probed with affinity-purified anti-ssrA-DD tag antibody (WB). Immunocomplexes were detected by ECL. D. Tagging in *S. lividans* analyzed as in panel C.

To distinguish the roles of *S. coelicolor* tmRNA in ribosome recycling function and in degradation of tagged proteins, we created a derivative of *ssrA*, modeled after well-characterized mutants in other organisms, in which the codons encoding the C-terminal two Ala residues of the tmRNA tag are altered to encode Asp [10], [15], [26]. To do so, a segment of the *ssrA* gene was replaced with a synthetic duplex DNA that altered not only the Ala codons but also complementary bases predicted to participate in a stem structure such that the stability of this structural element would not be perturbed (Figure 1A). Northern blot analysis using a probe that does not distinguish between endogenous wild-type tmRNA and the *ssrA-DD* derivative, demonstrated that the total steady state amount of *ssrA* tmRNA was approximately doubled in cells with a single extra-copy and was substantially higher in cells transformed with the multi-copy plasmid (Figure 1B).

Based on previous experiments by others we anticipated that the addition of the altered tag via *trans*-translation would result in the accumulation of tagged polypeptides that would not be recognized or degraded by the ClpXP and other proteases. To detect these products, polyclonal antibodies were raised in rabbits using a synthetic peptide immunogen corresponding to the C-terminal ten amino acids of the mutant tag with an N-terminal Cys residue to facilitate chemical crosslinking (NH_3_-CSSQQAFALDD-COOH). The antiserum was capable of detecting GFP tagged with a similar tag based on the sequence of *E. coli ssrA* (AANDENYALDD-COOH; data not shown) suggesting that the C-terminal four amino acids (ALDD-COOH) of the tag is enough for the recognition by these antibodies.

Western blotting with affinity-purified antibody revealed that lysates of wild type cells lacking the mutant *ssrA-DD* tmRNA displayed very little cross-reactivity (Figure 1C). Tagged proteins were readily detected in cells with a single integrated copy of the mutant *ssrA-DD* expressed from its own promoter. A similar pattern of tagged proteins, but with a much more intense signal, was detected in cells in which mutant tmRNA was overexpressed from a multi-copy plasmid. The fact that tagging by the mutant tmRNA can be readily detected even in the presence of the wild type *ssrA* gene suggests that the mutant tmRNA is at least competent to compete with its wild type counterpart for ribosome recycling. As a further test of the function of the mutant *ssrA-DD* construct we also expressed it in *S. lividans* 66, whose tmRNA sequence is identical. In a previous report [25] tagging by a similarly modified tmRNA was detectable only when the tmRNA was overexpressed along with a model tmRNA target protein in an *ΔssrA* background. In contrast, we were able to easily detect tagging of endogenous proteins in *S. lividans* even in a wild type background with an intact wild type *ssrA* gene (Figure 1D).

### Influence of *bldA* on tmRNA tagging

**Figure 2:**
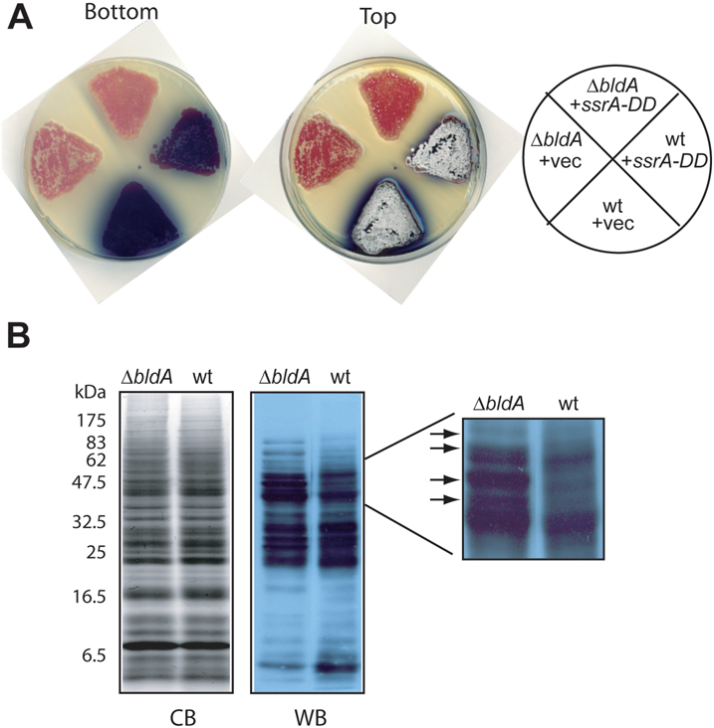
Effect of *bldA* expression on tmRNA-mediated tagging. A. Developmental phenotypes were determined for wild type (wt) and Δ*bldA* strains either transformed with a high copy-number vector with no insert (+vec) or with the same vector containing *ssrA-DD* (+*ssrA-DD*). Plates were scanned from the top and bottom to illustrate both spore and pigment production. B. Western blot analysis of the strains carrying *ssrA-DD* described in A was performed as described in the legend to Figure 1. Arrows highlight tagged bands that are more readily detected in the Δ*bldA* cells in the magnified image.

UUA codons decoded by the Leu-tRNA^UUA^ derived fromn the *bldA* gene occur rarely in the G+C-rich *S. coelicolor* genome. Because ribosome stalling at rare codons is known to elicit tmRNA tagging [10] we reasoned that a strain lacking the *bldA*-encoded tRNA might display an altered pattern of tagged proteins in the presence of the *ssrA-DD* allele. We first determined that overexpression of mutant tmRNA did not alter the phenotype (delayed pigment formation and absence of aerial mycelia and spores) of the Δ*bldA* strain (Figure 2A). Although analysis of the *S. coelicolor* genome predicts that 145 open reading frames contain UUA codons [18], we observed relatively few changes in the pattern of tagged proteins (Figure 2B). In the majority of mRNAs containing UUA codons, the UUA codons are found near the 5′-end of the open reading frame suggesting that tmRNA-mediated tagging at UUA codons could give rise to a number of short polypeptides [27]. Nonetheless, the extra immunoreactive bands observed in extracts of the Δ*bldA* strain (see blow up in Figure 2B) were in the ∼40 to 60 kDa range.

### Resistance of the *ssrA* gene to disruption at high temperature

**Figure 3:**
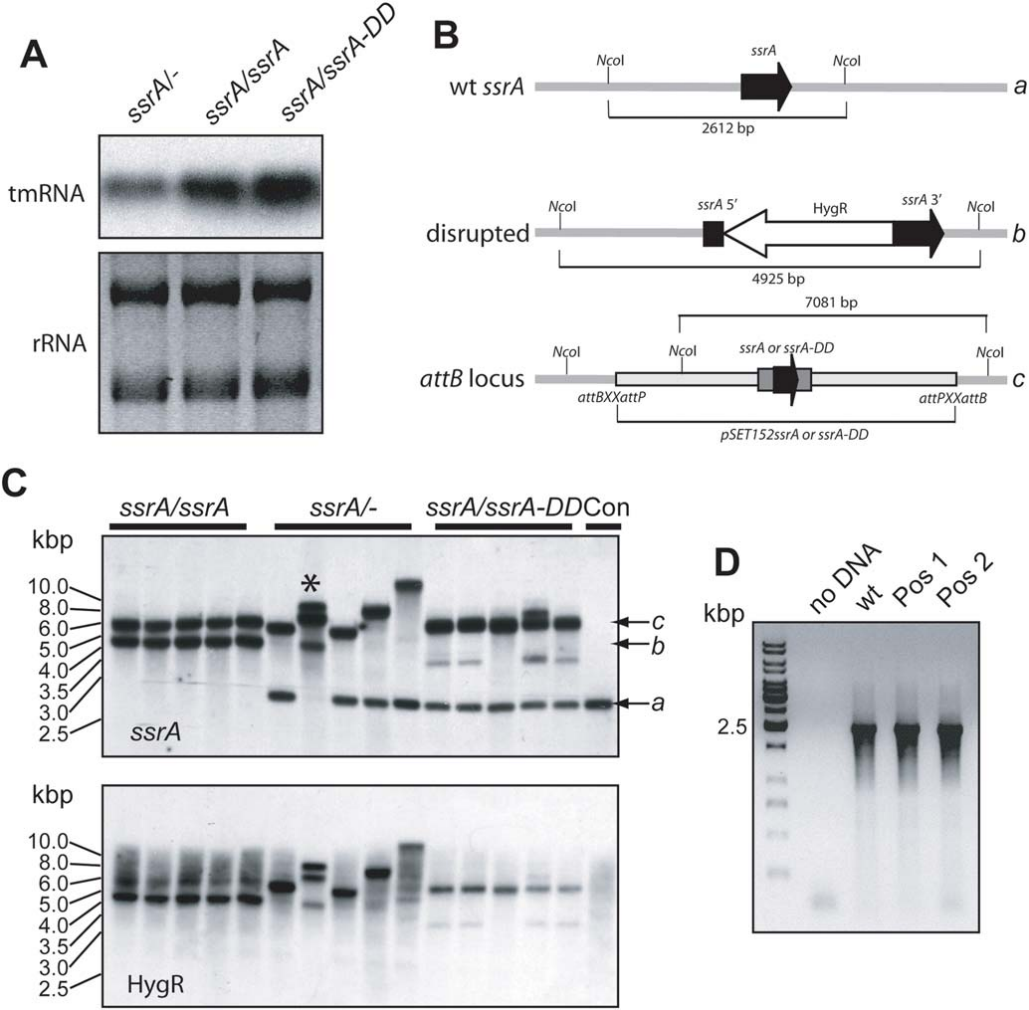
Insertional mutagenesis using a temperature-sensitive replicon. A. Northern blot analysis. Total RNAs were extracted from *S. coelicolor* strains with marker only, wild type *ssrA* or *ssrA-DD* inserted at the *attB* locus and analysed by Northern blot using a probe that does not distinguish between wild type and mutant tmRNA. Ethidium bromide stained ribosomal RNA (rRNA) is shown as a loading control. B. Schematic diagrams illustrating the anticipated genomic maps of the *ssrA* locus, the *attB* locus following integration of *ssrA* or *ssrA-DD*, and the *ssrA* locus following correct integration of the HygR cassette. The expected sizes of *Nco*I restriction fragments are indicated in each case. Lower case letters to the right of each fragment indicates fragment labeled in panel C. C. Southern blot analysis of *Nco*I-digested genomic DNA isolated from “positive” strains that were resistant to hygromycin and sensitive to thiostrepton. Ten isolates from each group were analyzed, five of which are illustrated. The top Southern blot was probed with an *ssrA* probe while the bottom blot was hybridized with a probe for the HygR cassette after the same blot was stripped. Control lane (Con) is genomic DNA from an untransformed wild type strain. Arrows and letters indicate genomic fragments illustrated in Panel B. D. Two of the ten positives from the *ssrA*/- group with an unusual Southern blot result (one of these is indicated by asterisk in panel B) were further analyzed by genomic PCR of the *ssrA* region with genomic DNA template from wild type cells used as a control. An ethidium bromide-stained agarose gel is illustrated.

To determine what role tmRNA might play in *S. coelicolor* growth and development we constructed three strains in which to carry out disruption of the genomic *ssrA* locus. One of these (*ssrA/ssrA*) carried an additional copy of the *ssrA* gene inserted into the φ31 locus using the integrating plasmid pSET152 [28] while a second strain (*ssrA/ssrA-DD*) carried the *ssrA-DD* derivative at the same locus. The third strain (*ssrA/-*) contained only the apramycin resistance marker from pSET152 inserted at the φ31 locus. tmRNA expression from the integrated extra-copy of either *ssrA* or *ssrA-DD* was confirmed by Northern blot analysis (Figure 3A). As anticipated, both extra-copy strains accumulated higher levels of tmRNA than the control strain with only a single *ssrA* gene.

All three strains were transformed with the temperature-sensitive plasmid pGM160 [29] carrying a hygromycin (HygR) resistance cassette [30] flanked by about 1 kb of *ssrA* sequence and a thiostrepton (ThiR) resistance marker. After initial selection on medium containing both hygromycin and thiostrepton, spores from transformants were pooled and grown in liquid culture at 39°C for 48 h during which time pGM160-based plasmids should fail to replicate. To obtain single colonies, ground mycelia from liquid cultures were plated and sporulated at 39°C. Hygromycin-containing plates were seeded with spores and the resulting colonies were replica plated onto medium containing thiostrepton. Among the population of hygromycin resistant colonies, positives were scored as colonies that were now sensitive to thiostrepton indicating plasmid loss and probable genomic integration of the HygR cassette.

We anticipated that if *ssrA* disruption were lethal, positive colonies would be most abundant in the strain with an extra-copy *ssrA*. In fact the majority of the transformed *ssrA/ssrA* pseudodiploids were scored as positives (Table 1). However the single copy *ssrA/-* strain yielded a low but significant percentage of positives as did the *ssrA/ssrA-DD* pseudodiploid. To determine the rate of occurrence of false positives in these populations of selectants, Southern blot analysis was performed on a collection of ten colonies of each of the three groups (Figure 3C shows the results from five isolates for each strain). We anticipated the possible outcomes of *Nco*I digestions of genomic DNA with the expected sizes of different loci shown in Figure 3B. In all strains derived from the *ssrA/ssrA* pseudodiploid, an *ssrA* probe detected *Nco*I fragments corresponding to the extra copy *ssrA* gene and the normal *ssrA* disrupted with the HygR. In *ssrA/-* cells the wild type genomic *ssrA* fragment was detected in eight of the ten strains analyzed (Figure 3C). In the two strains that did not have a band corresponding to the wild type genomic *ssrA*, PCR analysis of genomic DNA indicated that a product of the same size as that obtained for wild type genomic DNA was detected (Figure 3D) suggesting that these cells may have undergone some genomic reorganization affecting one or more of the relevant *Nco*I sites flanking the *ssrA* gene. In the *ssrA/ssrA-DD* lines, all colonies analyzed had a genomic fragment corresponding to the wild type genomic *ssrA*. Collectively these analyses indicate that true positives with the cassette faithfully targeted to the wt genomic *ssrA* locus were detectable only in the *ssrA/ssrA* pseudodiploid while both the *ssrA*/*ssrA-DD* and *ssrA*/- were refractory to *ssrA* disruption and in every case retained the wild type *ssrA* gene.

**Table 1:** Occurrence of “positives” during insertional mutagenesis of *ssrA* using the temperature sensitive plasmid pGM160.

Genotype	Total hyg^R^	hyg^R^/thio^R^	% thio^S^ / hyg^R^
*ssrA/ssrA*	604	153	74.7
*ssrA/ssrA-DD*	1228	1019	17.0
*ssrA/-*	855	728	14.9

**Figure 4:**
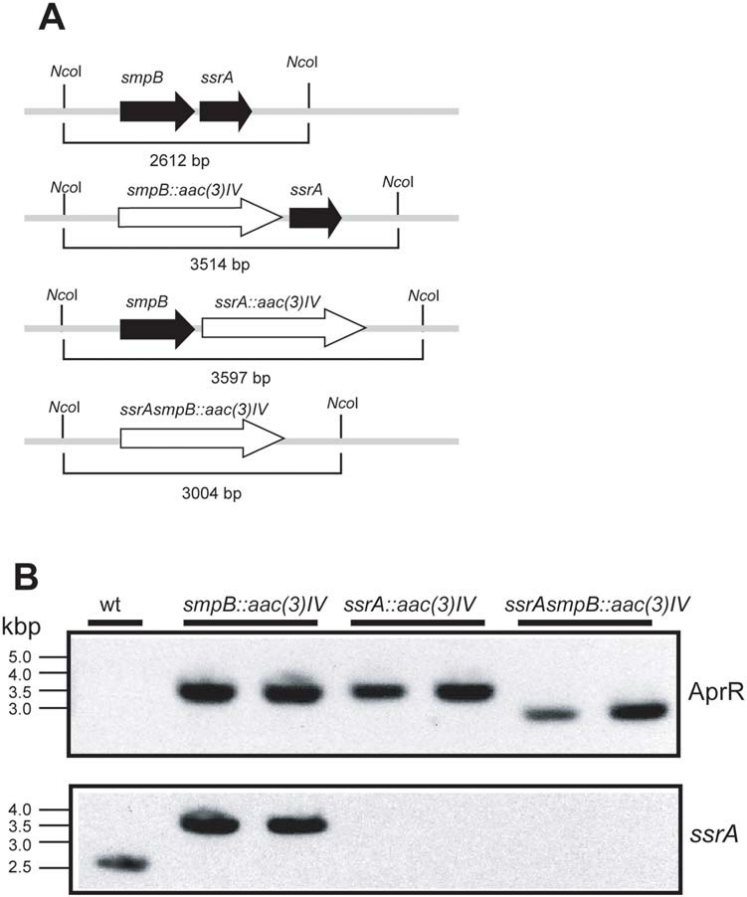
Southern blot analysis of *ΔsmpB*, *ΔssrA* and *ΔsmpB/ssrA* strains. A. Schematic diagrams of the genomic map of the wild type genomic *smpB/ssrA* locus and the anticipated structures following integration of the apramycin resistance cassette. The expected sizes of *Nco*I restriction fragments are indicated in each case. B. Southern blot analysis of *Nco*I-digested genomic DNA isolated from two independent isolates that were resistant to apramycin and sensitive to kanamycin. After detection with a probe for the aprR cassette (top), the blot was stripped and redetected with an *ssrA* probe (bottom).

Initially we concluded from these experiments that *ssrA* is essential for normal growth of *S. coelicolor* and that the *ssrA-DD* allele cannot substitute for wild type *ssrA* implicating an important role, not only for ribosome recycling, but also the degradation of tagged polypeptides. However, in contrast to these results others [25] reported the successful disruption of *ssrA* in *S. lividans*, a closely related species to *S. coelicolor*, using a cosmid-mediated mutagenesis technique [31]. We therefore repeated our effort to disrupt *S. coelicolor ssrA* using this methodology. Southern blot analysis was performed using probes corresponding to both apramycin resistance marker and *ssrA* gene with genomic DNA from two independent isolates for each disruption strain. *Nco*I fragments of genomic DNA (Figure 4A) detected with the apramycin resistance gene probe (AprR) corresponded to the sizes predicted for disrupted *ssrA*, *smpB* and *ssrA/smpB* disrupted together (Figure 4B; upper panel). The *ssrA* probe detected bands only in wild type and *smpB* deletion strains (Figure 4B; lower panel). Thus we conclude that the tmRNA system in *S. coelicolor* is not essential.

**Figure 5:**
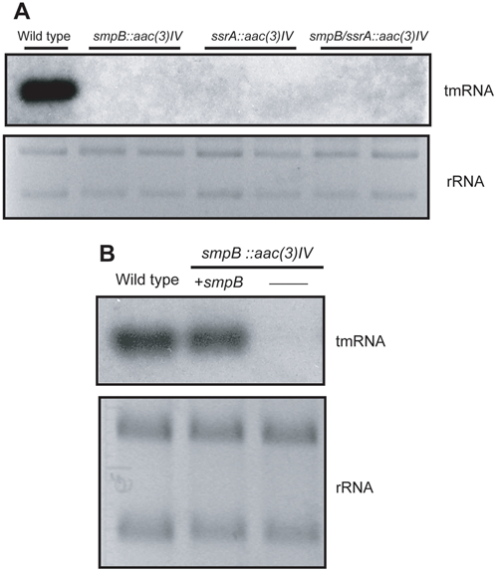
Northern blot analysis of the mutant strains. A. Total RNAs from the same isolates shown in Figure 4 were prepared and probed for tmRNA expression by Northern blot. An ethidium bromide-stained agarose gel showing rRNAs is illustrated as loading control. B. The *ΔsmpB* strain was transformed with an SmpB expression plasmid was compared by Northern blot analysis with control (wild type) and mutant cells without complementation.

To further characterize these strains, the expression of tmRNA was analyzed by Northern blot. As expected, tmRNA was not detected in Δ*ssrA* and Δ*smpB*/Δ*ssrA* mutants (Figure 5A). No tmRNA was detected in the *ΔsmpB* mutant either even though the *ssrA* gene was untouched in this strain. When a similar observation was made in an Δ*smpB* strain of *S. lividans* it was postulated that deletion of the *smpB* gene which is situated immediately upstream of the *ssrA* gene, exhibited a polar effect on the transcription of *ssrA* [25]. In our case, transformation of the Δ*smpB* strain with an SmpB expression plasmid restored the accumulation of tmRNA (Figure 5B) supporting the idea that the absence of tmRNA in the *ΔsmpB* strain can not be attributed to perturbed transcription of *ssrA* but rather demonstrates that SmpB protein is crucially required for tmRNA accumulation. This is consistent with promoter analysis that demonstrates the majority of tmRNA is transcribed from its own promoter (Yang and Glover, manuscript in preparation).

### tmRNA is important for *S. coelicolor* cell growth, development and resistance to stresses

**Figure 6:**
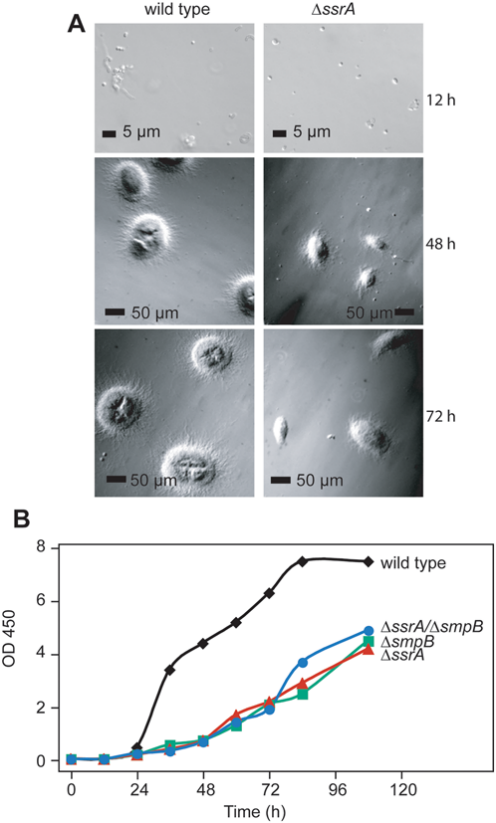
Liquid growth phenotypes of the mutant strains. A. *S. coelicolor* wild type (left) and Δ*ssrA* mutant (right) strains were grown in YEME medium at 30°C from spores. Phase-contrast micrographs were taken with a Zeiss microscope at 12 h, 48 h, and 72 h. B. Wild type and mutant strains were first grown up from spores in YEME medium in a 30°C shaker at 250 rpm until culture reached log phase (OD 450 = 1–2) and then ground manually to break up mycelium, diluted into fresh YEME medium to equal density of OD 450 at 0.05 and further cultured with shaking at 30°C.

When spores were inoculated into liquid medium and incubated at 30°C, we noted that wild type *S. coelicolor* formed a coarse suspension while cultures of Δ*ssrA* strains looked initially turbid. To examine this difference in detail we examined cultures microscopically. 12 h post-inoculation wild type spores had germinated and begun to form mycelia whereas Δ*ssrA* spores remained ungerminated (Figure 6A). After 48 h, wild type cells had formed substantial mycelial clumps whereas Δ*ssrA* clumps were smaller and the cultures contained loose mycelium as well as single cells and ungerminated spores. At 72 h, the size differential in mycelial clumps was more pronounced but both strains had begun to accumulate red pigment at the center of the clumps.

By examination of cultures alone it was difficult to determine if the differences in cultures were due primarily to defects in germination or also in growth of mycelia. To compare the growth rate of wild type and mutant cells, mycelia were harvested and dispersed by grinding and inoculated into fresh medium at equal density. Under this condition, the Δ*ssrA*, Δ*smpB* and the combined deletion (Δ*smpB*/Δ*ssrA*) strains all grew slower than the wild type strain (Figure 6B). These results suggest that the SmpB-tmRNA tagging system contributes both to spore germination and growth in liquid culture.

**Figure 7:**
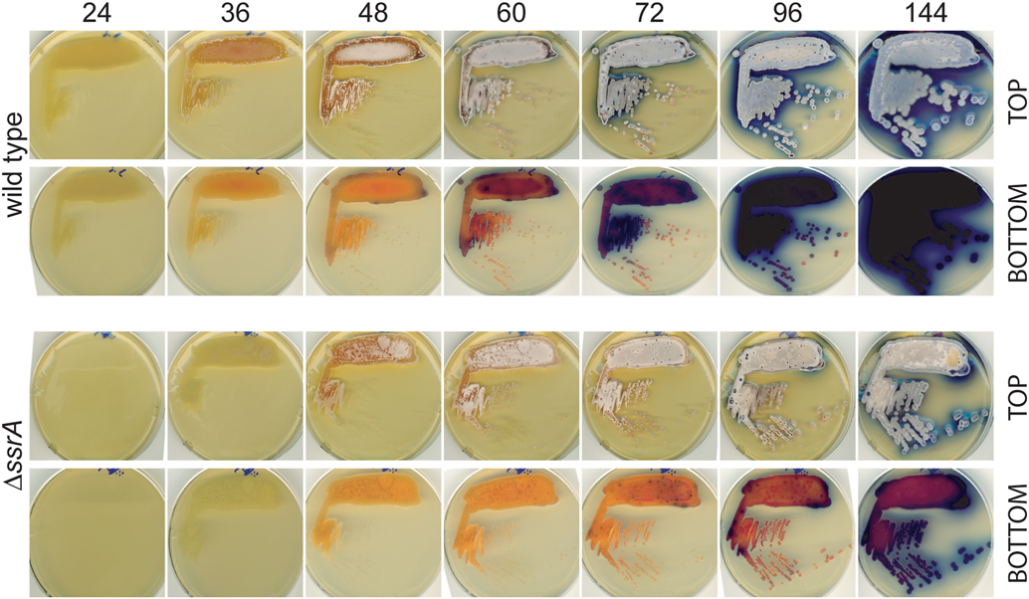
Agar growth and sporulation phenotypes of the mutant strains. Equal number (based on OD 450) of wild type and *ssrA* mutant spores were resuspended in 50 µL YEME medium, streaked onto the surface of R2YE plate and incubated at 30°C. At indicated time points, plates were scanned from both top and bottom of the plate to show surface growth and pigment production.

A similar delay is observed when spores are germinated on plates. In addition to delay in the formation of vegetative mycelia, the *ΔssrA* strain fails to accumulate blue pigment to the same extent as wild type cells even after prolonged incubation (Figure 7). Even though mutant cells form aerial mycelia and spores, we consistently found that the yield of spores was only about 1/5 that of wild type cultures after one or two weeks of growth. In addition, spores from mutant cultures were pink in liquid suspension in contrast to the black-colored wild type spore (not shown).

**Figure 8:**
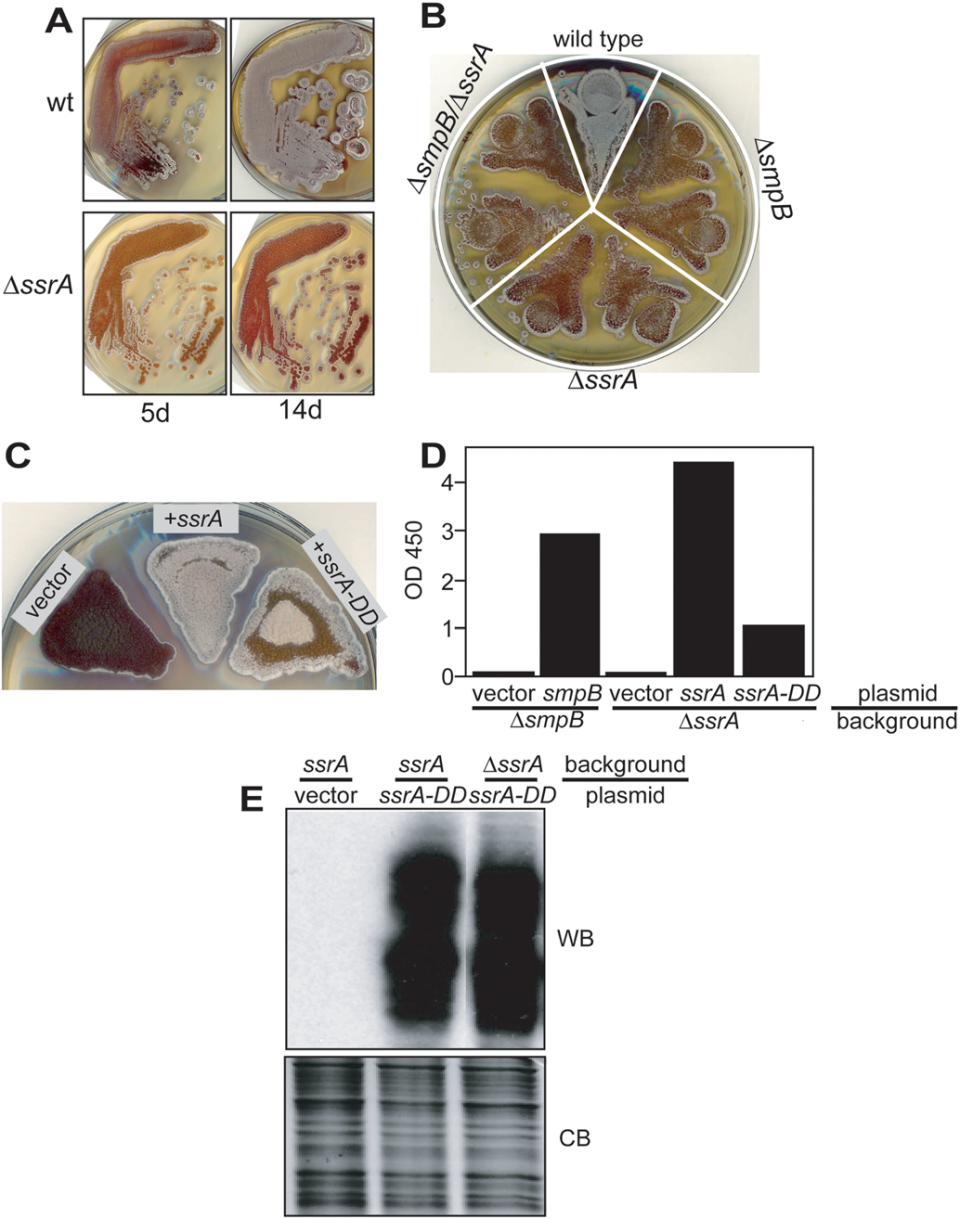
Phenotype at high temperature and mutant complementation. A. Wild type and mutant spores were plated as previously described in the legend to Figure 7 and grown at 39°C. At day 5 and 14, pictures were taken from the top to show growth and sporulation. B. Wild type and two independent isolates each of the indicated single and double mutants were first cultured in liquid YEME medium to log phase and equal number of cells (based on OD 450) were plated onto R2YE agar plate and grown at 39°C for 5 d. Plates were scanned from top. C. Spores of the Δ*ssrA* strain, both uncomplemented and complemented with *ssrA* or *ssrA-DD*, were cultured in YEME in a 250 rpm shaker at 30°C for 3 d and equal densities (based on OD 450) were plated onto R2YE agar plate and further incubated at 39°C for 5 d. D. Spores of Δ*smpB* and Δ*ssrA* strains with and without complementing genes were inoculated into YEME medium to an OD 450 of 0.03 and grown at 39°C for 5 d. OD 450 was measured and used to plot the graph. E. Wild type and Δ*ssrA* strains were transformed with a multicopy *ssrA-DD* plasmid and tagging was analyzed by Western blot as described in Figure 1C legend.

Since we were unable to obtain an Δ*ssrA* strain using the temperature-sensitive plasmid, we suspected that *ssrA* may be even more important for sporulation at the high temperatures used during this effort. Indeed, when the log phase cells were plated at 39°C, the Δ*ssrA* isolates were able to grow and form substrate mycelium but produced very limited aerial mycelium compared to the wild type control (Figure 8A) and this was also true of Δ*smpB* and Δ*smpB*/Δ*ssrA* strains (Figure 8B). Complementation of the Δ*ssrA* strain with wild type *ssrA* restored sporulation at 39°C while the *ssrA-DD* allele only partially corrected the sporulation defect (Figure 8C). In addition, liquid cultures inoculated with Δ*smpB* or Δ*ssrA* spores and incubated at 39°C barely grew compared to the same strains complemented with the corresponding wild type genes (Figure 8D). As observed in sporulation at 39°C, the *ssrA-DD* allele only partially restored high temperature growth in liquid culture. Taken together, these results provide a plausible explanation as to why we could only disrupt *ssrA* in the presence of a complementing extra-copy of the wild type allele and not in its absence or in the presence of the *ssrA-DD* allele that only partially complements the sporulation and germination defects exhibited by an Δ*ssrA* strain.

The lack of full complementation by the *ssrA-DD* allele cannot be explained by reduced expression of the mutant tmRNA relative to wild type because Northern blot analysis suggests that it is expressed at the expected level when present on a single- or multi-copy plasmid (see Figure 1B). Others have reported that mutant tmRNA is not as highly active as the wild-type tmRNA in tagging of proteins and recycling stalled ribosomes [8], [10], [32], [33] pointing to another plausible reason why *ssrA-DD* fails to fully complement Δ*ssrA* in germination and sporulation. We tested whether tmRNA^DD^ was substantially outcompeted for tagging by the wild type tmRNA by analyzing its tagging function in wild type and Δ*ssrA* backgrounds. By Western blot, we do not detect substantial differences in tagging (Figure 8E), supporting the idea that wild type tmRNA does not strongly compete with mutant tmRNA under the conditions used in our complementation experiments. This observation supports the idea that the degradation of tagged proteins is at least partially required for normal spore germination and sporulation at high temperature.

### *ssrA-DD* cannot rescue hygromycin sensitivity in a Δ*ssrA* strain

**Figure 9:**
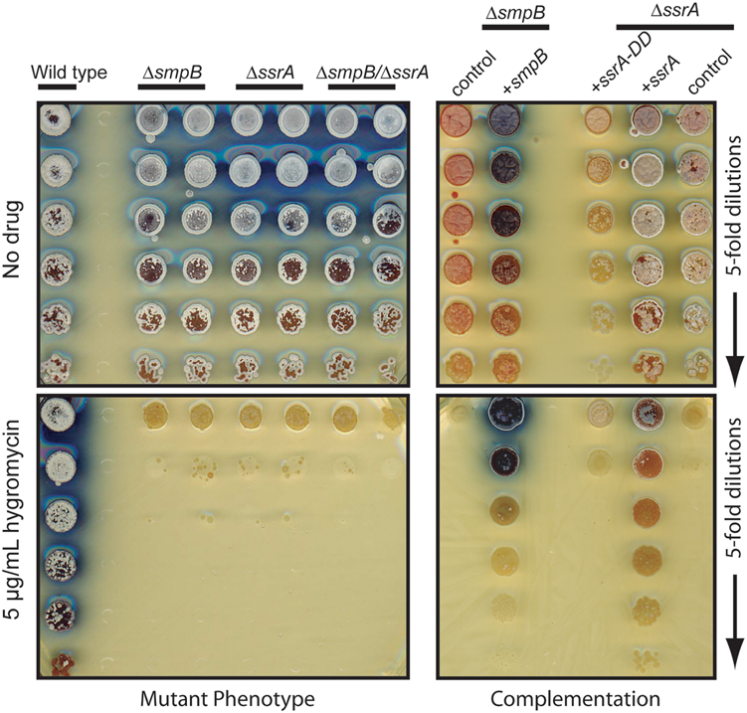
Complementation of hygromycin sensitivity. *Left panel*. Wild type and mutant spores (two independent isolates each) were serially diluted 5-fold and plated onto R2YE agar without any drug (upper) or with 5 µg mL^−1^ hygromycin (lower). *Right panel*. Spores of mutant cells with and without complementing genes were serially diluted and plated onto R2YE agar plate without drug (upper) or with 5 µg mL^−1^ hygromycin (lower). All plates were scanned from top after grown at 30°C for 5 d.

In addition to phenotypes observed at both normal and high temperatures, we also tested if mutant strains are more sensitive to antibiotics that interfere with translation elongation. Based on the reported cross-resistance conferred by the *aac(3) IV* construct encoding apramycin acetyltransferase [49], we expected that our mutant strains would tolerate a number of aminoglycoside antibiotics but not hygromycin. In the presence of a sublethal concentration of hygromycin, wild type cells grew well but the growth of strains lacking the SmpB-tmRNA tagging system were strongly inhibited (Figure 9). Both Δ*smpB* and Δ*ssrA* complemented with the corresponding wild type genes recover wild type hygromycin resistance. Even though *ssrA-DD* partially rescues the high temperature sporulation defect in *ΔssrA* strain, we found that it completely failed to rescue hygromycin resistance.

## Discussion

During translation elongation when ribosomes encounter a termination codon, the sequence of events (reviewed in [34]) that lead to polypeptide release and recycling of the ribosome is initiated by polypeptide release factors RF1 (UAG/UAA) or RF2 (UGA/UAA). After cleavage of the ester bond between the 3′ nucleotide of the P-site tRNA and the nascent polypeptide, RF1 or RF2 is removed from the ribosome A site in a GTP-dependent reaction involving RF3. RRF (ribosomal recycling factor) and EF-G (elongation factor G) then bind to the 70S post-termination complex and, in a GTP-requiring reaction, dissociate the post-termination complex into the 50S subunit and the 30S/mRNA/deacylated tRNA complex. To recycle the 30S subunit, the deacylated tRNA is removed from the P site by IF3 (initiation factor 3). The 30S subunit either dissociates spontaneously from the previously bound mRNA or scans the mRNA to resume translation at a nearby initiation site

While this sequence of termination events is important for the recycling of ribosomes, it is obstructed when the ribosomes stall at the ends of truncated mRNAs lacking a termination codon. Ribosomes may also stall at nonsense codons or weak termination codons, each lacking contextual features that elicit efficient termination. Under these circumstances, ribosome stalling can induce the cleavage of the mRNA by an unknown mechanism producing a non-stop mRNA [35], [36] that, in turn, becomes a target for the action of tmRNA [37]. The consequences of this action are three-fold. First, elongation and termination on the mRNA-like segment of tmRNA ensures the recycling of ribosomal subunits contributing to the maintenance of a healthy ribosomal pool. Second, degradation of tagged polypeptides released by the action of tmRNA ensures the timely removal of aberrant molecules that may interfere with normal cellular processes. Third, tmRNA facilitates the degradation of non-stop mRNAs [38], [39].

In this work we initially failed to disrupt *ssrA* in *S. coelicolor* using a strategy that employed a temperature sensitive replicon even though another nonessential gene (encoding a secreted triaminopeptidase) was easily knocked out (CY and JRG unpublished observation). We first suspected that *ssrA* gene might be essential in *S. coelicolor* and thus incorporated additional controls into our knockout experiment including extra copy of wild type and mutant *ssrA-DD*. Using the temperature-sensitive plasmid strategy, the *ssrA* gene was knocked out only when an extra-copy of wild type *ssrA* gene was present but not when the mutant *ssrA-DD* was inserted as an extra-copy allele into the genome. Because the deletion of *ssrA* in *S. lividans* was reported to have no strong phenotypic consequences [25] we could not imagine that the same gene deletion in such a closely related species would be lethal as suggested by our results.

After successfully knocking out the *ssrA* gene using the cosmid-mediated recombination methodology, we were able to demonstrate germination, growth, and sporulation defects of *ΔssrA* mutant at both high and routine growth temperatures. Therefore, the method used to promote integration of the temperature sensitive replicon and the production of spores to obtain pure strains would have significantly impeded the recovery of knockouts. We conclude that even though *ssrA* gene is not essential, it plays an important role during the *S. coelicolor* life cycle and that is even more crucial at high temperature. Our result is consistent with the recent discovery that tmRNA tagging is required for sporulation in *Bacillus subtilis*, another sporulating Gram-positive bacteria [40].

We were able to easily detect tagging of a number of endogenous proteins in both *S. coelicolor* and *S. lividans* when tmRNA^DD^ was expressed from a single-copy integrated gene or from a multicopy plasmid-borne gene. This result is remarkably different from that reported previously [25] in which tmRNA^DD^ tagging in *S. lividans* was only detectable when a reporter protein, APH (aminoside phospho transferase), was overexpressed from a nonstop mRNA in a *ΔssrA* strain overexpressing the modified *ssrA*-*DD* gene. We conclude that there is no significant species difference that can account for this discrepancy. The different results might be partially attributed to differences in the construction of tmRNA^DD^ derivatives. In our case, care was taken to not only alter the tag coding region but to include compensatory changes in complementary bases to preserve the folding stability of a large stem loop structure that includes much of the tag coding region. However, it is equally likely that our anti-ssrA-DD tag antibody is much more sensitive than that used in the previous report.

Altering the two C-terminal Ala residues of the tag may stabilize many polypeptides that would otherwise be rapidly degraded in the presence of the wild type tmRNA. However, altering the sequence of tmRNA at these codons may also impair tmRNA-facilitated non-stop mRNA degradation [38]. Under ideal conditions, defects that are complemented equally well by the wild type and the *ssrA-DD* alleles can be primarily attributed to impaired ribosome recycling in the absence of tmRNA. By comparison, defects that cannot be rescued by the *ssrA-DD* allele maybe dependent on the degradation of tagged proteins in wild-type strains, mRNA clearance, or both. However, the efficiency with which tmRNA^DD^ initiates *trans*-translation is thought to be significantly lower than that of wild type tmRNA in *E. coli* [8] thereby further complicating the simple interpretation of non-complementation. When tagging by tmRNA^DD^ was analyzed by western blot in our experiments, the presence of wild type tmRNA did not noticeably diminish tmRNA^DD^-mediated tagging suggesting that wild type tmRNA does not effectively out-compete tmRNA^DD^ for stalled ribosomes when the two alleles are present simultaneously. Thus we are confident that our tmRNA^DD^ can efficiently initiate *trans*-translation on stalled ribosomes and can provisionally attribute the hypersensitivity of *ΔssrA* cells to hygromycin to the accumulation of mistranslated polypeptides and/or non-stop mRNAs. We cannot exclude the possibility that accumulation of aberrant proteins bearing mutant tags have unanticipated negative effects on cell growth although we do not detect any growth defects when tmRNA^DD^ is overexpressed in wild type or Δ*bldA* cells.

Our *smpB* deletion was phenotypically identical to the deletion of *ssrA* and resulted in the reduction of tmRNA accumulation to below levels detectable by Northern blot. A similar result in *S. lividans* was attributed to polar effects caused by insertional mutation of the gene (*smpB*) immediately upstream of *ssrA* [25]. However, in our hands, expression of SmpB from a plasmid restores tmRNA accumulation in *ΔsmpB* strain demonstrating that *ssrA* transcription is not affected by *smpB* disruption. Rather this result is consistent with previous findings that SmpB binding plays a role in the stability of tmRNA in other organisms [41]–[44] although such a drastic effect has not been hitherto observed.

If indeed the degradation of tmRNA-tagged polypeptides is important then mutations in genes for components of the degradation machinery may also confer growth or developmental phenotypes, especially under stress conditions. In other organisms the majority of polypeptides tagged by *trans*-translation are degraded by the ATP-dependant ClpXP protease [4], [26]. But other proteases, including the related ClpAP protease, Tsp [7], [45] and Lon [46] can also be involved in the turnover of tmRNA-tagged proteins. Interestingly the disruption of *clpX* accelerates the time course of antiorhidin production in *S. lividans* [47] and impairs aerial mycelia formation on acidic medium [48]. The *S. lividans* genome is reported to have five ClpP-encoding genes. The disruption of *clpP1* in *S. lividans* blocks the formation of aerial mycelia [47] but also induces the transcription of genes encoding clpP3 and clpP4 [49]. Although these experiments implicate Clp protease-dependent processes in normal growth and development, it is likely that some of these effects are pleiotropic and so influence a number of cellular processes that are dependent on the turnover of other proteins in addition to those that are tmRNA-tagged.

Ribosome stalling at codons that are decoded by rare tRNAs are also known to be targets of the SmpB/tmRNA system [10]. Thus the tmRNA tagging system may intersect with *bldA*-controlled regulatory pathways to coordinate *Streptomyces* development. The abundance of *bldA*-encoded tRNA is low in early growth and development and reaches its highest level in stationary phase [50]. In agreement with the temporal control of the regulatory tRNA, accumulation of GFP expressed from a UUA-containing mRNA is delayed during early differentiation [51]. When *bldA*-encoded tRNA level is low, stalled ribosomes on UUA-containing mRNA may be targeted by the tmRNA system leading to degradation of prematurely terminated polypeptides and degradation of corresponding mRNA. In line with this idea, recent analyses of both the proteome and transcriptome in *S. coelicolor* provides evidence that levels of some UUA-containing mRNAs are reduced in the absence of *bldA* [52]. Although we uniquely detected a few proteins that are tagged in Δ*bldA* cells, we have no evidence that these specific are translated from UUA containing mRNAs. They may simply arise from the translation of mRNAs that are upregulated in Δ*bldA* and which are subject to tagging for other reasons.

UUA codons are found in many mRNAs encoding proteins involved in the regulation of antibiotic synthesis and cell development [53]. These include the mRNAs transcribed from the *actII-ORF4* and *redZ* genes encoding proteins that regulate the expression of genes involved in the biosynthesis of the two antibiotics conferring blue and red pigmentation in *Streptomyces* colonies, and the *adpA* genes needed for normal morphological development. It is possible that dynamic competition between the tmRNA tagging system and the *bldA* tRNA for ribosomes stalled at UUA codons contributes to post-transcritional regulation of the expression of rare-codon containing genes. If so, we would predict that elimination of tmRNA would favor accumulation of some regulatory proteins and their corresponding mRNAs even when *bldA* tRNA is low potentially accelerating pigment accumulation and transition from vegetative to sporulative growth. By extension, tmRNA overexpression might be predicted to delay these processes. In contrast to these predictions, we see a delay in development and pigmentation when tmRNA is eliminated and no discernable phenotype when tmRNA is over-expressed. Reduction of ribosome recycling in the Δ*ssrA* mutant may only marginally alter the production of efficiently translated, high abundance proteins, while severely impacting the accumulation of low abundance proteins, including regulatory proteins translated from mRNAs containing rare codons, and thereby contribute to the defects we observed. Since the *adpA* gene has been identified as a major contributor to the morphological deficiency of *bldA* mutants [21], [54], it would be interesting to determine if *adpA* mRNA is specifically targeted by the tmRNA tagging system and more generally, determine the identities of some of the tmRNA tagged proteins we observed in our experiments.

## Materials and Methods

### Strains and culture conditions

*S. coelicolor* strains M145 (prototrophic, SCP1^−^ SCP2^−^) [55] and mutant strains were grown on solid R2YE agar or in liquid yeast extract-malt extract (YEME) medium[55]. To test drug sensitivity, different concentrations of antibiotics were added into cooled R2YE agar before pouring. J1501 (*hisA1 uraA1 strA1 SCP1^−^ SCP2^−^*) [56] and its corresponding Δ*bldA* strain, J1681 were a gift from Brenda Leskiw [50].

### Amplification of *ssrA* and its flanking regions

Genomic DNA was prepared from *S. coelicolor* M145 mycelium grown in liquid YEME medium and used as a template for PCR. PCR was performed using the TaqPCRx GC-rich kit (Invitrogen) and the primers ssrA-1F and ssrA-1R (see Table 2 for primers and oligonucleotides used in this study). After 32 cycles (95°C, 30 s, 58°C, 30 s, 68°C 2 min) a ca. 1970 bp product containing both the *ssrA* tmRNA coding sequence, as well as 962 bp upstream and 618 bp downstream of *ssrA*, was cloned into pCR2.1-Topo (Invitrogen) and sequenced (ACGT Corp, Toronto). The resulting plasmid was named pCRTopossrA. For the description of all the plasmids used in this study, see Table 3.

**Table 2:** Oligonucleotides used in this work.

Name	Sequence (5′ – 3′)
ssrA-1F	CCGGCGGCGACACCACCAGA
ssrA-1R	TGTTCGGCGCCATGGAGGTCGTCA
ssrA-BglII	AGATCTGCAAGAAGGAGTACGACAA
ssrA-HindIII	AAGCTTCTGGACGGTGGAACACGTCT
ssrADD-F	GATCCGGGTCGGGAACAGCCCCGGGCTGACACTCCCTTAGTAGGAGGCTCGCTTCGACGACTGAGATCAGTCGTCGAGGGCGAAGG
ssrADD-R	CCTTCGCCCTCGACGACTGATCTCAGTCGTCGAAGCGAGCCTCCTACTAAGGGAGTGTCAGCCCGGGGCTGTTCCCGACCCG
PSmpB-PstI	CGCTGCAGATGGCTAAGGAAAAAGGGC
PSmpB-Rev	CGAAGCTTCAGGCCCGCTGCTTGCG
PSmpB59nt-5′	GCCAGGCCCCTCCGTAGTGCCAAAATGGACCGCACTATGATTCCGGGGATCCGTCGACC
PSmpB58nt-3′	GGACGCGTGCCAGCCTATTCCCGGCGGCGGGCGGCGTCATGTAGGCTGGAGCTGCTTC
PSsrA59nt-5′	GCGAGCCCCTCTTCGGAGGACTTGAAAAATCAACATGGGATTCCGGGGATCCGTCGACC
PSsrA58nt-3′	TCGCGCTTACGCGAGCACACGCCGCCCTCGACCTCGTGGTGTAGGCTGGAGCTGCTTC

**Table 3:** Plasmids used in this work.

Plasmid	Description	Source/reference
PCR2.1Topo	Cloning vector	Invitrogen
PCRTopossrA	2 kb DNA covering *smpB/ssrA* genomic region inserted into PCR2.1Topo	This work
pCAN46	*Streptomyces* protein expression vector containing *aph* promoter and signal peptide sequence (SP)	Ni et al., 2003
pCAN46Δ	pCAN46 deleted promoter and SP	This work
pCAN46ΔSsrA	*ssrA* gene and promoter region inserted into pCAN46Δ	This work
pCAN46ΔSsrA-DD	*ssrA-DD* gene and promoter region inserted into pCAN46Δ	This work
pCAN46aph	pCAN46 deleted SP only, still has the *aph* promoter	This work
pCAN46aphsmpB	SmpB coding sequence inserted into pCAN46aph	This work
pSET152	*Streptomyces* site-specific integrating vector	Bierman et al., 1992
pSET152SsrA	*ssrA* gene and promoter region inserted into pSET152	This work
pSET152SsrADD	*ssrA-DD* gene and promoter region inserted into pSET152	This work
pHP45Ωhyg	Shuttle plasmid containing the hygromycin resistance cassette	Blondelet-Rouault et al., 1997
PGM169	Unstable and temperature sensitive plasmid useful for gene replacement	Muth et al., 1989
PGM160SsrA::Hyg	*ssrA*::Hyg disruption cassette inserted into the pGM160 plasmid	This work
PIJ773	Containing disruption cassette with apramycin resistance marker	Gust et al., 2002

### Construction of plasmids

To overexpress tmRNA, the *ssrA* gene, with its 5′- and 3′-flanking regions, was amplified by PCR using primers ssrA-BglII and ssrA-HindIII. The resulting 829 bp amplified fragment was digested with *Bgl*II and *Hind*III and ligated to the multicopy, pIJ101-based overexpression plasmid pCAN46 [57] that was previously digested with the same enzymes. The recombinant plasmid, designated pCAN46Δ*ssrA*, has the original pCAN46 promoter and signal peptide-encoding sequence replaced by *ssrA* driven by its own promoter. To create a mutant *ssrA* gene in which the final two Ala codons of the tag sequence are converted to Asp codons, synthetic complementary oligonucleotides, ssrADD-F and ssrADD-R, were denatured at 96°C for 15 min, cooled slowly to allow annealing and ligated into *Bam*HI/*Stu*I-digested and gel purified pCAN46ΔssrA. The sequence of the inserted segment of the recombinant plasmid, designated pCAN46ΔssrA-DD, was verified by DNA sequencing.

To introduce a second copy of *ssrA* or *ssrA-DD* into the chromosome of *S. coelicolor* M145, the *ssrA* or *ssrA-DD* coding and flanking regions were removed from pCAN46ΔssrA and pCAN46ΔssrA-DD with *Bam*HI and *Hin*dIII, blunted with Klenow polymerase, and ligated to the *Eco*RV site of the φC31 *attP* site-integrating plasmid pSET152 [28] to form pSET152ssrA or pSET152ssrA-DD. To create a targeted disruption plasmid, the hygromycin resistant cassette was excised from pHP45Ωhyg [30] by *Bam*HI digestion, blunted and ligated to the *Stu*I site of pCRTopo-*ssrA* to form pCRTopossrA::Hyg. This plasmid was then digested with *Spe*I and *Xba*I to release the *ssrA*::Hyg construct for insertion into the *Xba*I site of the temperature-sensitive replicon, pGM160 [29] to create pGM160ssrA::Hyg.

To re-express SmpB in the Δ*smpB* mutant cells, the signal peptide-encoding segment of pCAN46 was deleted to form pCAN46aph containing only the *aph* promoter to drive downstream gene expression. The *smpB* coding region was amplified by PCR using pCRTopossrA as template and pSmpB-PstI and pSmpB-Rev as primers. The amplified DNA fragment was digested with *Pst*I and *Hin*dIII and ligated into the pCAN46aph backbone that has been previously digested with *Pst*I and *Hin*dIII to form pCANaphsmpB.

### Preparation of cell-free extracts, antibody and western blot analysis

To prepare cell-free extracts, mycelia were harvested and resuspended in P-buffer [58] containing 2 mg mL^−1^ lysozyme (Sigma). Mycelial suspensions were incubated at 30°C for 10 to 30 min until protoplasting was just detectable, at which time the mycelia were pelleted and resuspended in RIPA lysis buffer [59] before sequential passage through 20 and 22 gauge syringe needles. The lysates were cleared of cell debris by centrifugation at 16,000×*g* at 4°C for 10 min. The supernatant was transferred to a fresh tube and total protein concentration was measured with a detergent compatible protein assay reagent (Biorad). SDS-polyacrylamide gel electrophoresis (PAGE; 20% polyacrylamide) was performed with 20 µg of protein/lane on minigels (8 cm×5 cm×0.75 mm) and 60 µg protein/lane on larger gels (15 cm×14 cm×1 mm).

A peptide (CSSQQAFALDD) corresponding to the last 10 amino acids of the *ssrA-DD* tag with an N-terminal Cys residue was synthesized by Alberta Peptide Institute (University of Alberta, Canada) and used for antibody generation. Rabbits were immunized with a peptide-KLH conjugate. The peptide-specific antibodies were purified by affinity purification using peptide conjugated to AffiGel-10 (Biorad). Purified antibody was stored in aliquots in PBS containing 15% (v/v) glycerol at −80°C. Proteins separated by SDS-PAGE were electrophoretically transferred to PVDF membranes (Millipore). The aanti-SsrA-DD primary antibody was used at 1∶4000 dilution and the HRP-conjugated goat anti-rabbit IgG (Promega) was used as secondary antibody at 1∶5000 dilution.

### RNA extraction

Tri Reagent (Sigma) was used to isolate total RNA following the manufacturer's protocol with some modifications. *S. coelicolor* M145 was grown either in liquid YEME medium or in solid culture on the surface of cellophane membranes on R2YE agar plates. Mycelia were harvested from liquid culture centrifugation for 10 min at 3,500×*g*, and from solid culture by scraping with a spatula. In both cases, mycelia were then washed once with 10.3% sucrose, resuspended in P-buffer containing 2 mg mL^−1^ lysozyme [58], and incubated at 30°C until the suspension becomes turbid signaling the formation of protoplasts, usually within 10 to 30 min. EDTA and SDS were then added to final concentrations of 100 mM and 2% (w/v) respectively. An equal volume of Tri Reagent was added and the suspension was vortexed vigorously prior to incubation at room temperature for 5 min. Chloroform (1/10 volume) was added and the mixture was vortexed and incubated at room temperature for another 5 min. After centrifugation at 12,000×*g* for 10 min at 4°C the supernatant was transferred to a fresh tube and extracted with equal volume of phenol∶chloroform (1∶1) and centrifuged as before. The aqueous phase was precipitated with an equal volume of isopropanol, washed once with 70% ethanol, dried at room temperature for 5 min, and then dissolved in DEPC-treated water. RNA concentrations were estimated by measuring OD at 260 nm.

### Northern blot analysis

To analyze the expression level of the *ssrA*-encoded tmRNA, 4 µg aliquots of total RNA were electrophoresed at 7 V cm^−1^ for 4 h on 1.8% (w/v) agarose gels containing 0.7% formaldehyde. The separated RNAs were then blotted overnight to Hybond-N^+^ membrane (AP Biotech) by capillary transfer using 10×SSC [60]. To make the probe, an 835 bp DNA fragment corresponding to *ssrA* and short flanking regions was amplified by PCR using pCAN46ssrA as template and ssrA-BglII, ssrA-HindIII as primers. PCR was performed at 95°C, 30 s; 56°C, 30 s; 68°C 1 min for 35 cycles. The amplified DNA was labeled with the AlkPhos Direct non-radioactive labeling kit (AP Biotech). Hybridization, membrane washing, and signal detection were performed following the manufacturer's protocol. To normalize the band density, RNA gels were stained with ethidium bromide before blotting and images were recorded with the GelDoc 2000 system (BioRad) to visualize the 23S and 16S rRNAs. After hybridization, the signals were detected either by the VersaDoc Imaging System (Model 5000, BioRad) or using Kodak film with suitable exposures.

### Southern blot analysis

Wild-type and mutant derivatives of *S. coelicolor* J1501 were inoculated into 15 mL YEME medium and the cultures were grown for 2 to 3 d. Mycelia were harvested and genomic DNA was prepared using the Kirby mix procedure [58]. Aliquots of 10 µg total DNA were digested to completion at 37°C with *Nco*I and electrophoresed on a 1% agarose gel for ∼4 h at 10 V/cm. After transfer to Hybond-N^+^ membrane (AP Biotech) the DNA was detected with the same *ssrA* fragment as described for Northern blot analysis. Hybridization, stringent washing, and detection procedures were performed according to the manufacturer's recommendations. The blots were also stripped and re-probed with non-radioactively labeled DNAs corresponding to HygR DNA released from pHP45ΩHyg [30] by *Hin*dIII digestion or AprR DNA released from pIJ773 [31] by *Eco*RI and *Hin*dIII digestion.

### Insertional inactivation of *ssrA*-pseudodiploid strategy

*S. coelicolor* M145 protoplasts were transformed with one of pSET152, pSET152*ssrA* or pSET152*ssrA-DD* and simultaneously with pGM160*ssrA*::Hyg. After overnight growth on R2YE plates, transformants were overlaid with 20 µg mL^−1^ apramycin to select for pSET152 and its derivatives, and with 12.5 µg mL^−1^ thiostrepton to select for pGM160*ssrA*::Hyg. After 3 to 4 d, resistant colonies were pooled and grown to high density in YEME containing 20 µg mL^−1^ apramycin and 10 µg mL^−1^ hygromycin B at 30°C. Mycelia were then spread onto R2YE plates containing apramycin and hygromycin B and the cultures were sporulated at 30°C. Spores were then harvested from the R2YE plate cultures, germinated and recultured to high density in liquid YEME containing apramycin and hygromycin B at 39°C to induce loss of the temperature-sensitive pGM160 replicon. After growth for 4 d at 39°C, the mycelia were re-spread onto R2YE plates containing apramycin and hygromycin B and grown at 39°C until the colonies developed spores. The harvested spores were diluted and plated onto R2YE plates (50 to 200 spores per plate) containing apramycin and hygromycin B and incubated at 39°C. Sporulated colonies were replica plated onto separate plates containing either 12.5 µg mL^−1^ thiostrepton or 10 µg mL^−1^ hygromycin B and incubated at 30°C for 2 to 3 d. From each plate, colony numbers were counted and the ratio of thiostrepton-sensitive/hygromycin B-resistant colonies was calculated.

### Disruption of *smpB*, *ssrA*, *smpB*+*ssrA*-Redirect technology

Disruption of *smpB*, *ssrA*, and *smpB+ssrA* is performed following the protocols of the “PCR-targeting system in *S. coelicolor*” developed by John Innes Center [31]. Cosmid E59 containing *smpB* and *ssrA* genes and surrounding sequences and other plasmids and bacteria strain were all obtained from John Innes Center. Upstream and downstream primers for both *smpB* and *ssrA* genes were designed following the protocol and PCR was performed using pIJ773 that contains the apramycin-resistant gene as template. To disrupt *smpB*, primers pSmpB59nt-5′ and pSmpB58nt-3′ were used, to disrupt *ssrA*, primers pSsrA59nt-5′ and pSsrA58nt-3′ were used, to disrupt both *smpB* and *ssrA* at the same time, primer pSmpB59nt-5′ and pSsrA58nt-3′ were used to amplify the corresponding extended apramycin-resistance cassette that is used to target the *S. coelicolor* cosmid E59. The mutant cosmids containing disrupted genes were transferred into *S. coelicolor* through conjugation by the non-methylating *E. coli* ET12567. Apramycin-resistant double cross-over exconjugants were selected and purified by streaking for single colonies and confirming kanamycin sensitivity and further verified by PCR and Southern blot analyses.
